# Beyond stroke therapy, neuroaid (a chinese herbal) has an effect on cognition and neurogenesis, a bibliometric study

**DOI:** 10.12688/f1000research.152581.1

**Published:** 2024-07-16

**Authors:** ARMAN YURISALDI SALEH, Riezky Valentina, Tirta Darmawan Susanto, Dwi Arwandi Yogi Saputra

**Affiliations:** 1 Neurology Department Faculty of Medicine, UPN Veteran Jakarta, Jakarta, Special Capital Region of Jakarta, 12450, Indonesia; 2 Family Medicine and Primary Care, Universitas Pelita Harapan, Tangerang, Banten, 15811, Indonesia; 3 Department of Public Health Sciences, Faculty of Medicine, UPN Veteran Jakarta, Jakarta, Special Capital Region of Jakarta, 12450, Indonesia

**Keywords:** neuroaid, cognitive enhancement, stroke, neuroprotective, bibliometric

## Abstract

**Introduction:**

NeuroAiD, also known as MLC601 or MLC901, is a Chinese herbal combination used worldwide for stroke treatment. It contains herbal components and five hewan components. MLC601 contains herbal components and hewan components, while MLC901 has a similar herbal composition. NeuroAiD is used to support neurologic recovery after stroke and to aid cognitive function in Alzheimer’s disease. Studies show that NeuroAiD has potential in treating Alzheimer’s disease and is beneficial in both local and global stroke models and in the Kortikal culture. However, there is limited bibliometric research on NeuroAiD, which is a method of collecting data from published articles to analyze developments and trends in the field of research. This research contributes significantly to the literature and helps develop more effective stroke treatment strategies.

**Methods:**

In this work, a literature review methodology is employed to gather data from the Scopus database using the keywords neuroaid. Data were analyzed using Biblioshiny and VOSviewer software to produce visualizations and bibliometric maps. We conducted quantitative and qualitative analysis

**Results:**

The research trend found are documents by year, most relevant sources, factorial map of the most cited documents, factorial map of The documents with the highest contributes, documents by author, documents by country or territory, documents by subject area, documents by affiliation, network visualization, overlay visualization of scopus database using vosviewer, density visualization, thematic map, thematic evolution, topic dendogram, and world cloud.

**Conclusions:**

The study investigates the potential of Neuroaid, a neuroprotective drug, for stroke prevention and cognitive function enhancement. It uses terms like “cognition” and “neurogenesis” to highlight its potential. While the study’s focus may be limited, it provides valuable insights into research direction and potential areas of neuroaid for stroke treatment.

## Introduction

NeuroAiD, also known as MLC601 or MLC901, is a combination of traditional Chinese herbs that has been used widely throughout the world for stroke therapy. ^1^^,^^2^ NeuroAiD contains nine herbal plants that help in blood circulation. ^1^

NeuroAiD (MLC601 and MLC901) is a drug derived from traditional Chinese medicine. Both formulations have herbal components, but there are differences in their composition.

### MLC601 (NeuroAiD)

MLC601 consists of nine herbal components and five animal components. The herbal components include: Radix astragali, Radix salviae miltiorrhizae, Radix paeoniae rubra, Rhizoma chuanxiong, Radix angelicae sinensis, Carthamus tinctorius, Prunus persica, Radix polygalae, and Rhizoma acori tatarinowii.

Animal components in MLC601 include Hirudo, Eupolyphaga seu steleophaga, Calculus bovisartifactus, Buthus martensii, and Cornu saigae tataricae. ^3^

### MLC901 (NeuroAiD II)

MLC901 is a simplified version of MLC601 and contains only the same nine herbal components as MLC601. ^1^^,^^4^

Both forms have demonstrated neuroprotective and neurodegenerative effects in cerebral ischemia models. ^3^^,^^5^ However, MLC901 has several advantages that may improve patient compliance. ^6^ Although MLC601 and MLC901 have similar safety and efficacy profiles, MLC901 does not contain animal components, which makes it more acceptable to patients who may have objections to the use of animal products. ^6^ This drug has been used extensively to facilitate neurological recovery after stroke, especially in the non-acute phase. Additionally, NeuroAiD has also been used to support cognitive function in Alzheimer’s disease.

Clinical and preclinical studies suggest that NeuroAiD has a role in Alzheimer’s disease. Additionally, NeuroAiD has demonstrated neuroprotective and neuroregenerative properties in animal models of focal and global ischemia as well as in cortical cell cultures. These properties are critical in developing treatment strategies to reduce long-term disability from stroke, heart attack, and other brain injuries.

Although NeuroAiD has been widely researched, no bibliometric studies have been conducted using documents from www.scopus.com. Bibliometric studies are a research method that uses data from scientific publications to analyze developments and trends in a particular research field. In this context, bibliometric studies can provide valuable insight into NeuroAiD research and how its use has evolved over time.

This research is especially important as there is increasing research on NeuroAiD and an increasing number of neurology centers using NeuroAiD for stroke therapy. Apart from that, this research also aims to look at the possibility of using NeuroAiD as a standard stroke therapy in the future. Thus, this study hopes to make a significant contribution to the existing literature and assist in the development of more effective stroke treatment strategies.

## Methods

Bibliometric research is a research method that uses scientific publication data to describe and analyze the development of a field of science. This research aims to identify and map trends, patterns, and relationships between scientific documents related to certain topics. In this research, the topic chosen was neuroaid. This research uses data from the website www.scopus.com, which is one of the largest and most trusted databases for scientific publications. This research was conducted on early May 2024.

To carry out bibliometric research, the steps to follow are as follows:

1. Determine search keywords. In this research, the keywords used are neuroaid. These keywords are entered into the search column on the www.scopus.com site by selecting the topic field (title, abstract, keywords).
2. Filter search results. In this study, Were not filtered.
3. Download search results data. In this research, search result data is downloaded in three different formats, namely:
  • CSV (comma-separated value), which contains basic information about the document, such as title, author, affiliation, year, source, abstract, and keywords.
  • RIS (research information system), which contains detailed information about a document, including the references cited by the document.

Data for this study were collected by one reviewer. Reviewers work independently in collecting data from each report. To ensure data accuracy and clutter. In addition, we use automated tools, namely the *VosViewer* and *Biblioshiny* applications, to assist in the data collection and analysis process."

in this study we found are documents by year has been an increase in the number of documents; until 2023, there were 9 documents, most relevant sources is journal of cerebrovascular diseases, documents by author with the most authors with 30 documents are Venketasubramanian, N., documents by country or territory is singapore is the country with the largest number of document producers 37 documents, documents by subject area, network visualization, overlay visualization of scopus database using vosviewer, density visualization, thematic map based on the title shows that the niche theme is the keyword mitochondria signaling pathway, effects neuroprotective effect, dan oxidative stres toxicity, thematic evolution, cluster analysis, qualitative analysis, and word cloud

### Data collection

We used the following terms to do a search on the Scopus website, taking into consideration that this website contains research that is considered to be valid: TITLE – ABS – KEY (neuroaid) are the titles of the products that are under consideration. Ninety-two documents were received by us. We then save the document from Scopus in the form of a file with the extension.csv file following this step.

### Data analysis

Both the Biblioshiny and Vosviewer software packages were utilised in the analysis process.

### Quantitative analysis

*Documents by year*

**Figure 1.**
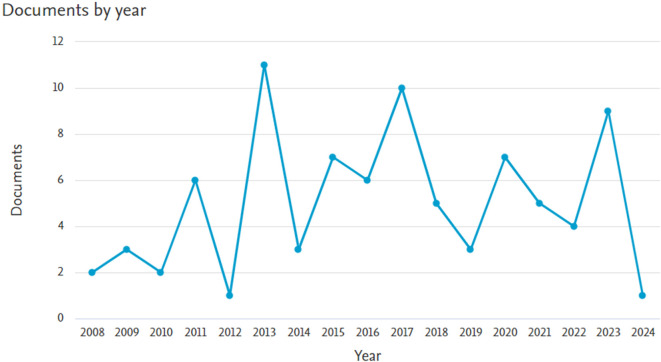
Documents by year.

Based on Figure 1, it appears that there has been an increase in the number of documents, until in 2023 there were 9 documents. The oldest document in 2008 was entitled Neuroaid in stroke recovery., written by Siow, C.H.C., ^7^ Next is the article entitled Danqi Piantan Jiaonang does not modify hemostasis, hematology, and biochemistry in normal subjects and stroke patients written by Gan, R. et al. ^8^ Meanwhile, the latest document in 2024 is entitled Facilitation of neurological recovery in a complete spinal cord injury with NeuroAiD: case report written by Zainudin, M.F., Abu Hassan, S.A., Khin, N.Y. ^9^

*Most relevant sources*

**Figure 2.**
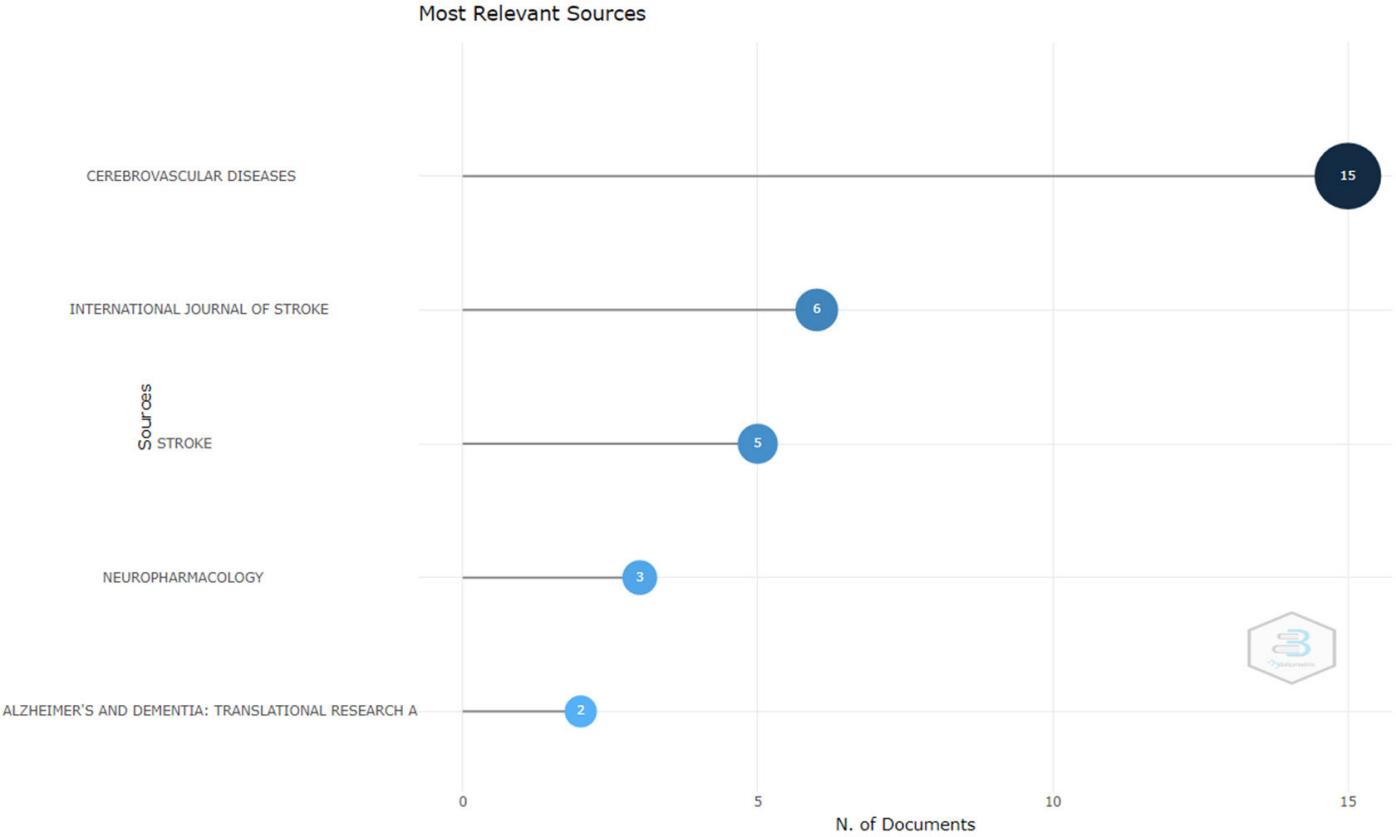
Most relevant sources.

Based on Figure 2, The following are the journals that publish the most important documents: The first is the Journal of Cerebrovascular Diseases. This journal is indexed in Scopus and is published by Karger Publisher. The SJR (SCImago Journal Rank) of this journal for 2023 is around 0.25. In 2023, this journal will be in the Q2 quartile for the cardiology and cardiovascular medicine category. The head office of Karger Publishers is located in Switzerland. The journal accepts manuscripts on topics covering prevention, diagnosis, management, rehabilitation, and related fields of molecular genetics, vascular biology, pathophysiology, epidemiology, and health systems science. The journal welcomes original research, reviews, and commentary articles focused on improving knowledge and information about clinical practice and health policy. The review time for this journal until the first decision is around 16.2 days. The H-index of this journal is 119. And the impact factor of this journal is around 2.96 in 2023.

The next journal is the International Journal of Stroke. This journal is indexed in Scopus and published by SAGE Publications Ltd. The SJR (SCImago Journal Rank) for 2023 is around 1,952. As of 2023, the journal is in the Q1 quartile for the categories “Neurology” and “Neurology (clinical). The head office of SAGE Publications is located in the United Kingdom. The journal accepts manuscripts on topics covering clinical aspects of stroke from around the world with basic scientific contributions in the field of clinical interest. This journal accepts reviews of current topics, leading opinions, research, panoramas, clinical trial protocols, and guidelines. The time for this journal’s review to the first decision is around 15.0 days from this journal, which is around 6,948 in 2023.

The next journal is the Stroke Journal. This journal has been indexed in Scopus since 1970. This journal is published by the American Heart Association. The SJR (SCImago Journal Rank) for 2023 is around 2,746. In 2023, this journal will be in the Q1 quartile for the categories Advanced and Specialized Nursing, Cardiology and Cardiovascular Medicine, Medicine (miscellaneous), Neurology (clinical), and Neuroscience (miscellaneous). The headquarters of the American Heart Association are located in the United States. The journal accepts manuscripts on topics covering all aspects of cerebral circulation and its diseases from many disciplines, including neurology, internal medicine, radiology, nuclear medicine, neuropathology, neurosurgery, epidemiology, vascular surgery, rehabilitation, anesthesiology, critical care, vascular physiology, neuropsychology, speech pathology, and neuro-ophthalmology. The H-index of this journal is 357. And the impact factor of this journal is around 10.17 in 2023.

*Factorial map of the most cited documents*

**Figure 3.**
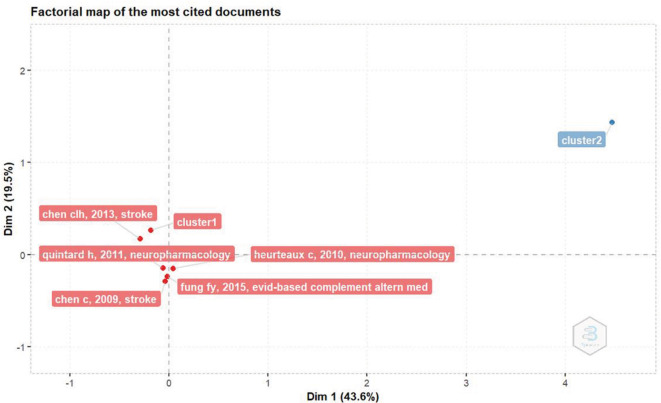
Factorial map of the most cited documents.

Based on Figure 3, the document with the most citations based on factorial analysis is The NeuroAiD II (MLC901) in Vascular Cognitive Impairment Study (NEURITES) by Chen CLH, Ikram K, Anqi Q, Yin WT, Chen A, and Venketasubramanian N, which was published in Cerebrovasc Dis in 2013. ^10^^,^^11^

This article discusses the NEURITES study, a 24-week, double-blind, randomized, placebo-controlled phase II study of NeuroAiD II in patients with vascular cognitive impairment (VCIND). The aim of this study was to investigate the effects and tolerability of NeuroAiD II in patients with VCIND. ^11^

NeuroAiD is a traditional Chinese medicine that has been shown to induce neuroplasticity, stimulate cell proliferation, and stimulate the development of dense axial and dendritic tissue in animal stroke models. NeuroAiD may be able to improve cerebral blood flow and functional recovery after stroke in patients. ^11^

The main outcome of this study was executive function as measured by the verbal fluency test. Secondary outcomes include cognitive assessments such as ADAS-Cog, MoCA, MMSE, and Cognitive Battery; daily activities as measured by the Alzhei-Mer’s Disease Cooperative Study Activities of Daily Living (ADCS-ADL) scale for mild cognitive impairment; behavior as measured by the Neuropsychiatric Inventory; and depression as measured by the Geriatric Depression Scale and Beck Depression Scale. ^11^

The NEURITES study has the potential to set new standards for the systematic evaluation of traditional Asian medicines for integration into standard medical practice and establish new therapeutic approaches to improve cognition after stroke. ^11^

*Factorial map of the documents with the highest contributes*

**Figure 4.**
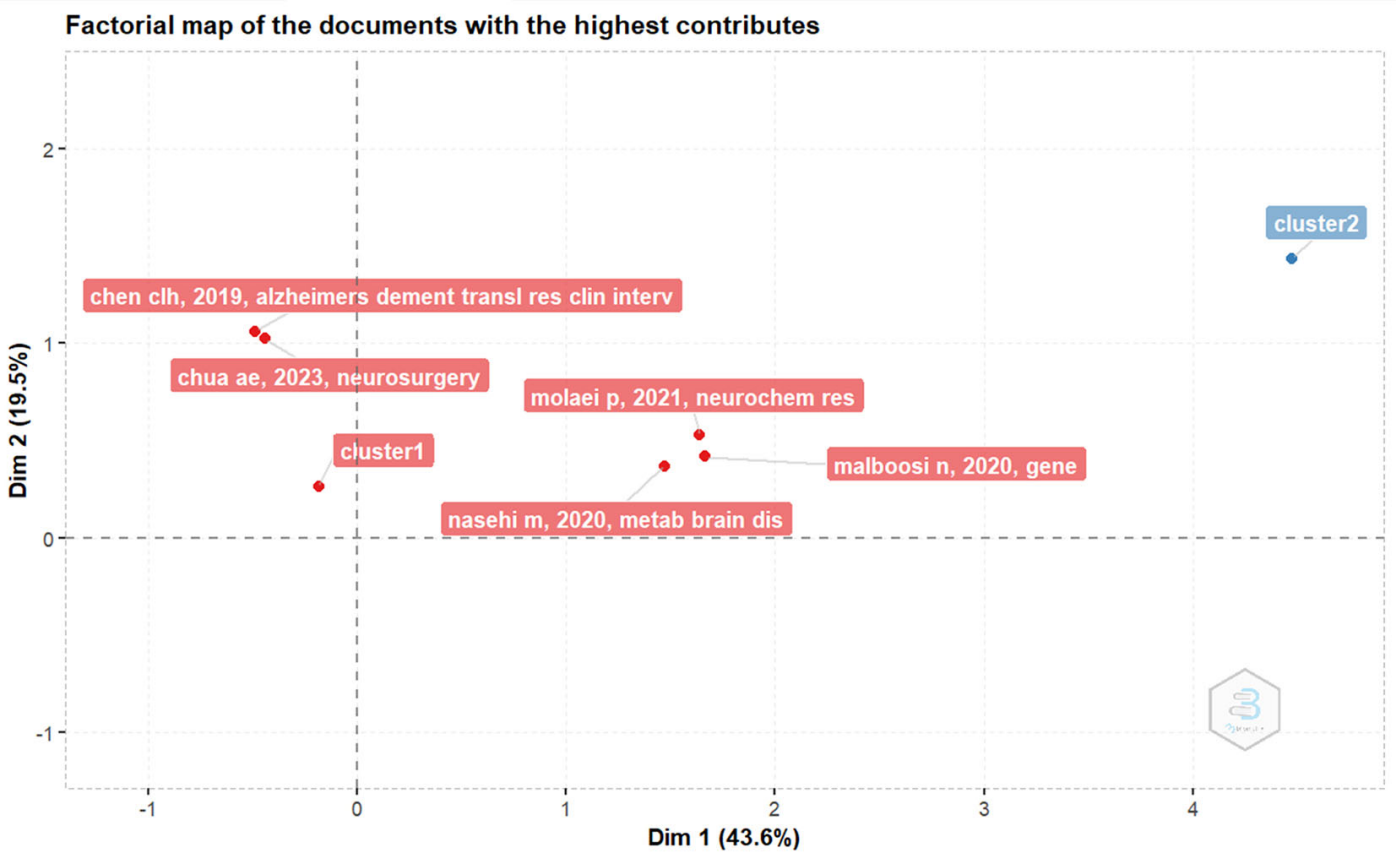
Factorial map of the documents with the highest contributes.

In Figure 4, the document with the highest contributor is the document entitled The Effect of NeuroAid (MLC901) on Cholestasis-Induced Spatial Memory Impairment with Respect to the Expression of BAX, BCL-2, BAD, PGC-1α, and TFAM Genes in the Hippocampus of Male Wistar Rats by Molaei P, Vaseghi S, Entezari M, Hashemi M, and Nasehi M, published in Neurochem Res, volume 46, pages 2154-2166, on May 24, 2021. ^12^

*Documents by author*

**Figure 5.**
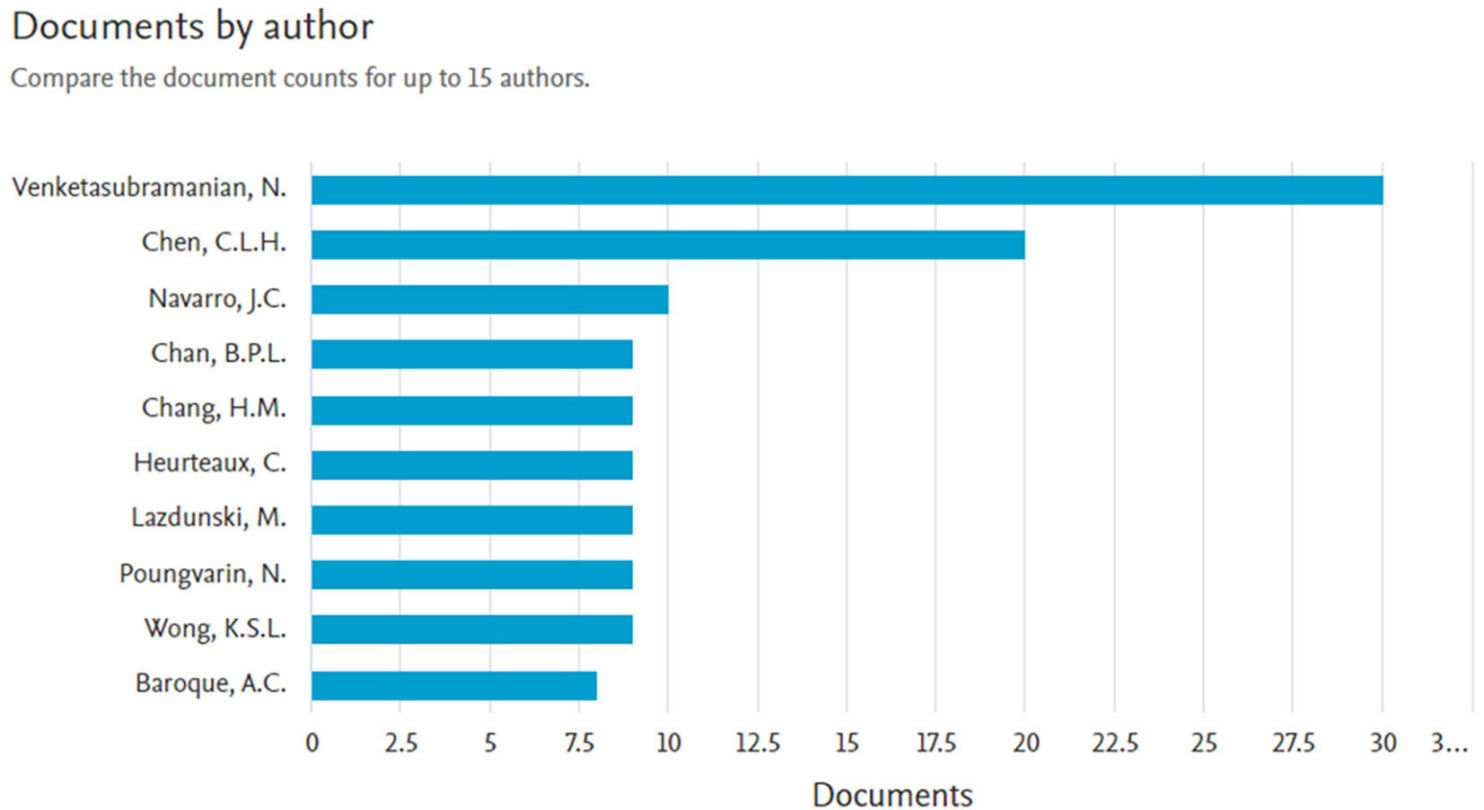
Documents by author.

Based on Figure 5 the most authors with 30 documents are Venketasubramanian, N., with the titles of several articles, namely: T Association between Baseline NIHSS Limb Motor Score and Functional Recovery after Stroke: Analysis Based on a Multicountry Dataset, ^13^ Ischemic Stroke and Savings in Time to Achieve Functional Recovery: Experience from NeuroAiD, ^14^ Alzheimer’s Disease THErapy With NEuroaid (ATHENE): A Randomized Double-Blind Delayed-Start Trial, ^15^ NEURoaid II (MLC901) in cognitively Impaired not demenTEd patientS (NEURITES): A pilot double blind, placebo-controlled randomized trial, ^16^ and NeuroAid II (MLC901) in Haemorrhagic Stroke. ^17^

The next most author with 20 documents is Chen, C.L.H., with the titles of several articles, namely: Association between Baseline NIHSS Limb Motor Score and Functional Recovery after Stroke: Analysis Based on a Multicountry Dataset, ^13^ Ischemic Stroke and Savings in Time to Achieve Functional Recovery: Experience from NeuroAiD, ^14^ Alzheimer’s Disease THErapy With NEuroaid (ATHENE): A Randomized Double-Blind Delayed-Start Trial, ^15^ NEURoaid II (MLC901) in cognitively Impaired not demenTEd patientS (NEURITES): A pilot double blind, placebo-controlled randomized trial, ^16^ and article title Frequency and clinical impact of serious adverse events on post-stroke recovery with NeuroAiD (MLC601) versus Placebo: The CHInese medicine neuroaid efficacy on stroke recovery study. ^18^

*Documents by country or territory*

**Figure 6.**
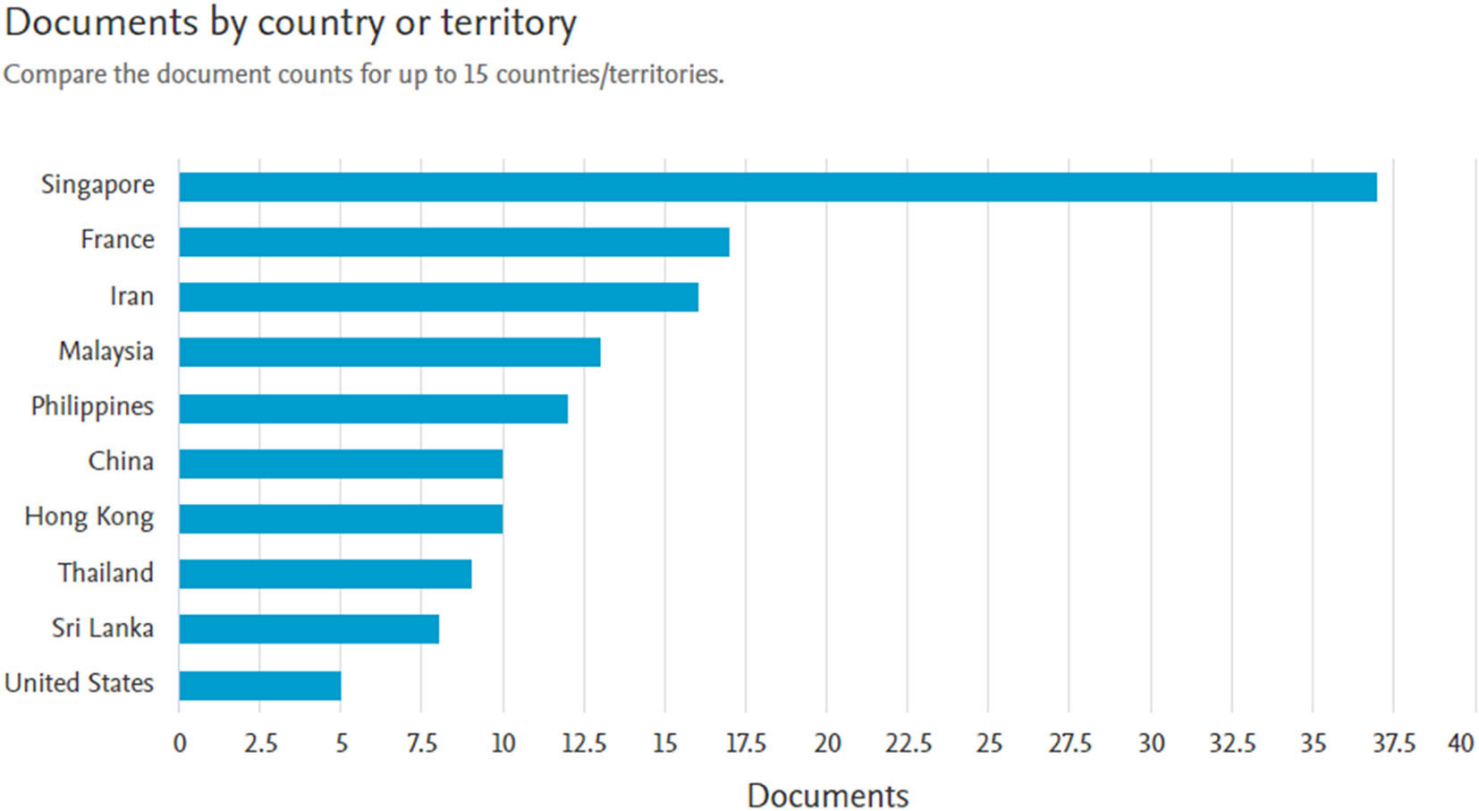
Documents by country or territory.

Based on Figure 6, Singapore is the country with the largest number of document producers 37 documents. Followed by France with 17 documents, Iran with 16 documents, Malaysia with 13 documents, and the Philippines with 12 documents.

*Documents by subject area*

**Figure 7.**
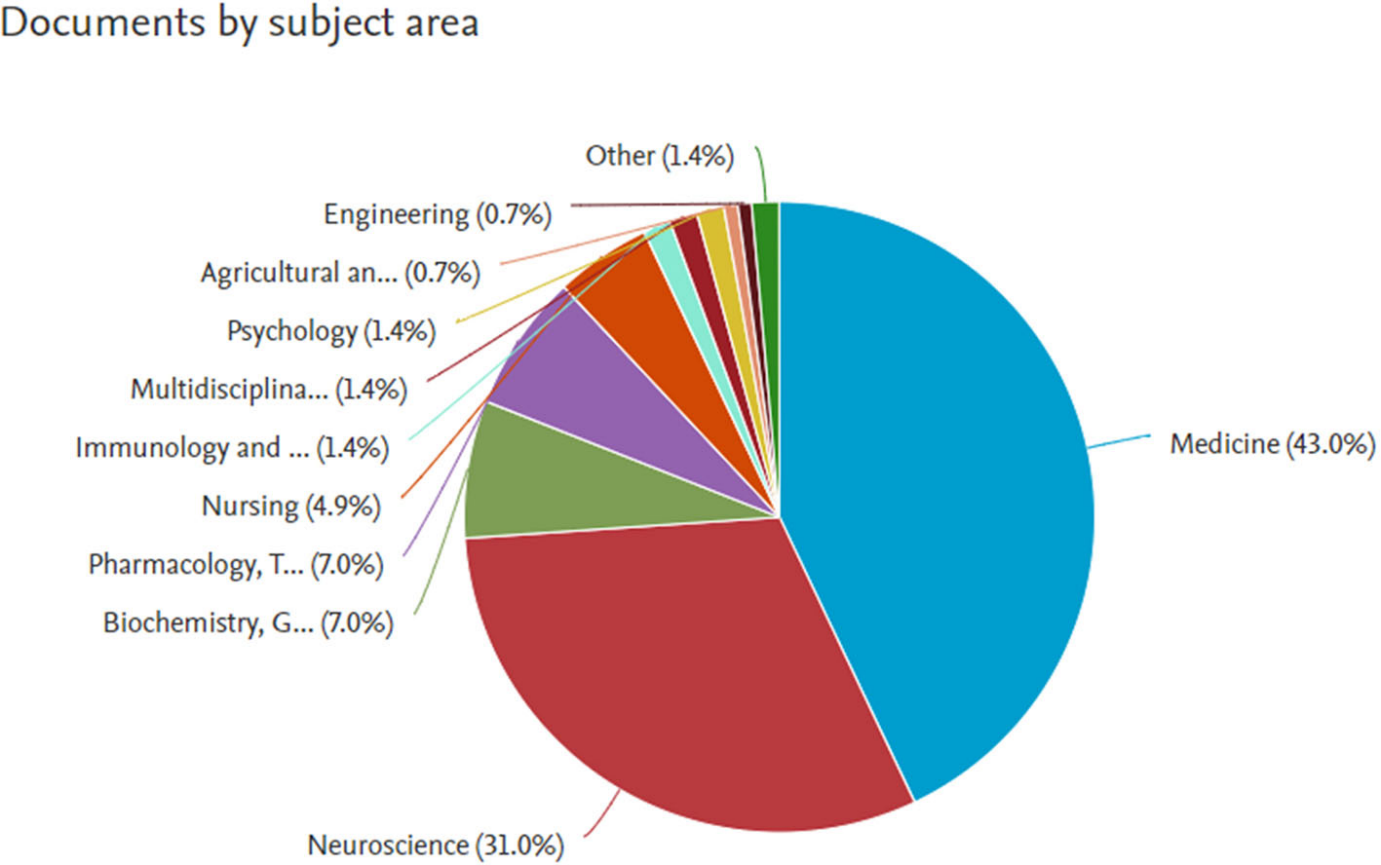
Documents by subject area.

**Table 1.** Documents by subject area.

No	Title
**Field of Medicine**	
1.	Facilitation of neurological recovery in a complete spinal cord injury with NeuroAiD: case report
2.	Protocol for Safety and Efficacy of MLC901 (NeuroAiD II) in Patients With Moderate Traumatic Brain Injury: A Randomized Double-Blind Placebo- Controlled Trial (ANDROMEDA)
3.	Brain computed tomography perfusion analysis in HIV-seropositive adults with and without neurocognitive impairment in Nigeria: outcomes and challenges of a pilot study
4.	MLC901 in hypoxic-ischemic brain injury patients: A double-blind, randomized placebo-controlled pilot study
5.	Association between Baseline NIHSS Limb Motor Score and Functional Recovery after Stroke: Analysis Based on a Multicountry Dataset
6.	Ischemic Stroke and Savings in Time to Achieve Functional Recovery: Experience from NeuroAiD
7.	A Long-term Study of NeuroAid (MLC601, MLC901) in Patients with Alzheimer's Disease; An Extension 8- year Follow-up Study
8.	INCOG 2.0 Guidelines for Cognitive Rehabilitation Following Traumatic Brain Injury, Part II: Attention and Information Processing Speed
9.	Spinal cord injury–assessing tolerability and use of combined rehabilitation and NeuroAiD (SATURN) study–primary results of an exploratory study
10.	Clathrin-nanoparticles deliver BDNF to hippocampus and enhance neurogenesis, synaptogenesis and cognition in HIV/neuroAIDS mouse model
11.	Randomised, double-blind, placebocontrolled study investigating Safety and efficAcy of MLC901 in posttraUmatic bRAin Injury: The SAMURAI study protocol
12.	Alzheimer's Disease THErapy With NEuroaid (ATHENE): A Randomized Double-Blind Delayed-Start Trial
13.	NeuroAid II (MLC901) and polypharmacy in stroke and the risk of hepatotoxicity: a case report
14.	NEURoaid II (MLC901) in cognitively Impaired not demenTEd patientS (NEURITES): A pilot double blind, placebo-controlled randomized trial
15.	Systematic Review and Meta-Analysis of the Efficacy of MLC901 (NeuroAiD II) for Acute Ischemic Brain Injury in Animal Models
16.	NeuroAid II (MLC901) in Haemorrhagic Stroke
17.	Role of MLC901 in increasing neurogenesis in rats with traumatic brain injury
18.	Frequency and clinical impact of serious adverse events on post-stroke recovery with NeuroAiD (MLC601) versus Placebo: The CHInese medicine neuroaid efficacy on stroke recovery study
19.	Effect of cholestasis and NeuroAid treatment on the expression of Bax, Bcl-2, Pgc-1α and Tfam genes involved in apoptosis and mitochondrial biogenesis in the striatum of male rats
20.	The Alzheimer's disease THErapy with NEuroaid (ATHENE) study protocol: Assessing the safety and efficacy of Neuroaid II (MLC901) in patients with mild-to-moderate Alzheimer's disease stable on cholinesterase inhibitors or memantine—A randomized, doubleblind, placebo-controlled trial
21.	Effect of Combined Treatment with MLC601 (NeuroAiD) and Rehabilitation on Post-Stroke Recovery: The CHIMES and CHIMESE Studies
22.	MLC901 (NeuroAiD II™) for cognition after traumatic brain injury: a pilot randomized clinical trial
23.	New directions in treatments targeting stroke recovery
24.	Efficacy and Safety of MLC601 in Patients with Mild to Moderate Alzheimer Disease: An Extension 4- Year Follow-Up Study
25.	Therapeutic effect of Chinese herbal medicines for post stroke recovery
26.	MLC601 in vascular dementia: An efficacy and safety pilot study
27.	Neuroaid for improving recovery after ischemic stroke
28.	Intracyctic papillary carcinoma of the breast: Report of a rare case and literature review
29.	Durability of the beneficial effect of MLC601 (NeuroAiD™) on functional recovery among stroke patients from the Philippines in the CHIMES and CHIMES-E studies
30.	Neuroaid for improving recovery after ischaemic stroke
31.	Efficacy and safety of MLC601 in the treatment of mild cognitive impairment: A pilot, randomized, double-blind, placebo-controlled study
32.	Prognostic factors and pattern of long-term recovery with MLC601 (NeuroAiD™) in the Chinese medicine NeuroAiD efficacy on stroke recovery - Extension study
33.	Sex and the treatment effect in the Chinese Medicine NeuroAiD Efficacy on Stroke recovery (CHIMES) trial
34.	MLC901 Favors Angiogenesis and Associated Recovery after Ischemic Stroke in Mice
35.	Efficacy of MLC601 on functional recovery after stroke: A systematic review and meta-analysis of randomized controlled trials
36.	Comparison between the original and shortened versions of the national institutes of health stroke scale in ischemic stroke patients of intermediate severity
37.	The value of patient selection in demonstrating treatment effect in stroke recovery trials: Lessons from the CHIMES study of MLC601 (NeuroAiD)
38.	CHInese medicine NeuroAiD efficacy on stroke recovery - Extension study (CHIMES-E): A multicenter study of long-term efficacy
39.	Therapeutic Efficacy of Neuro AiD™ (MLC 601), a Traditional Chinese Medicine, in Experimental Traumatic Brain Injury
40.	The NeuroAiD Safe Treatment (NeST) Registry: A protocol
41.	Prognostic factors and treatment effect in the CHIMES study
42.	Developing traditional Chinese medicine in the era of evidence-based medicine: Current evidences and challenges
43.	A Randomized trial to assess the long-term safety of NeuroAiD among Caucasian patients with acute ischemic stroke
44.	Effects of MLC601 on early vascular events in patients after stroke: The CHIMES study
45.	Chinese medicine neuroaid efficacy on stroke recovery: A double-blind, placebo-controlled, randomized study
46.	Drugs from natural substances: Why study them in cerebral infarction Bousser, M.-G. Cerebrovascular Diseases 2 View at Publisher Related
47.	NeuroAiD (MLC601) and amyloid precursor protein processing
48.	Chinese medicine NeuroAiD efficacy stroke recovery - Extension study (CHIMES-E study): An observational multicenter study to investigate the longer-term efficacy of NeuroAiD in stroke recovery
49.	NeuroAiD: Properties for neuroprotection and neurorepair
50.	Efficacy and safety of MLC601 (NeuroAiD®), a traditional chinese medicine, in poststroke recovery: A systematic review
51.	The NeuroAiD II (MLC901) in vascular cognitive impairment study (NEURITES) for the NEURITE investigators
52.	Cognitive enhancers (Nootropics). Part 3: Drugs interacting with targets other than receptors or enzymes. Disease-modifying drugs
53.	Alternative therapies for stroke treatment in Asia
54.	The effect of NeuroAiD™ (MLC601) on cerebral blood flow velocity in subjects' post brain infarct in the middle cerebral artery territory
55.	Safety and efficacy of MLC601 in Iranian patients after stroke: A double-blind, placebo-controlled clinical trial
56.	Use of Traditional Chinese Medication for post-stroke recovery
57.	Safety profile of MLC601 (Neuroaid®) in acute ischemic stroke patients: A Singaporean substudy of the chinese medicine neuroaid efficacy on stroke recovery study
58.	A double-blind, placebo-controlled, randomized phase II pilot study to investigate the potential efficacy of the traditional Chinese medicine neuroaid (MLC 601) in enhancing recovery after stroke (TIERS)
59.	Danqi Piantang Jiaonang (DJ), a traditional Chinese medicine, in poststroke recovery
60.	Neuroaid in stroke recovery
61.	Danqi Piantan Jiaonang does not modify hemostasis, hematology, and biochemistry in normal subjects and stroke patients
**Field of Neuroscience**	
1.	Facilitation of neurological recovery in a complete spinal cord injury with NeuroAiD: case report
2.	Association between Baseline NIHSS Limb Motor Score and Functional Recovery after Stroke: Analysis Based on a Multicountry Dataset
3.	NeuroAid II (MLC901) and polypharmacy in stroke and the risk of hepatotoxicity: a case report
4.	The Effect of NeuroAid (MLC901) on Cholestasis-Induced Spatial Memory Impairment with Respect to the Expression of BAX, BCL-2, BAD, PGC-1α and TFAM Genes in the Hippocampus of Male Wistar Rats
5.	A possible neuroprotective property of ethanol and/or NeuroAiD on the modulation of cognitive function
6.	Safety and use of MLC601/MLC901 (NeuroAiD™) in primary intracerebral hemorrhage: A cohort study from the NeuroAiD safe treatment registry
7.	Frequency and clinical impact of serious adverse events on post-stroke recovery with NeuroAiD (MLC601) versus Placebo: The CHInese medicine neuroaid efficacy on stroke recovery study
8.	Effect of cholestasis and NeuroAid treatment on the expression of Bax, Bcl-2, Pgc-1α and Tfam genes involved in apoptosis and mitochondrial biogenesis in the striatum of male rats
9.	The potential of mlc901 (Neuroaid ii™), a traditional chinese medicine
10.	Effect of Combined Treatment with MLC601 (NeuroAiD) and Rehabilitation on Post-Stroke Recovery: The CHIMES and CHIMESE Studies
11.	MLC901 (NeuroAiD II™) for cognition after traumatic brain injury: a pilot randomized clinical trial
12.	MLC901 (NeuroAiD II™) for cognition after traumatic brain injury: a pilot randomized clinical trial
13.	MLC601 in vascular dementia: An efficacy and safety pilot study
14.	Durability of the beneficial effect of MLC601 (NeuroAiD™) on functional recovery among stroke patients from the Philippines in the CHIMES and CHIMES-E studies
15.	Efficacy and safety of MLC601 in the treatment of mild cognitive impairment: A pilot, randomized, double-blind, placebo-controlled study
16.	Prognostic factors and pattern of long-term recovery with MLC601 (NeuroAiD™) in the Chinese medicine NeuroAiD efficacy on stroke recovery - Extension study
17.	The effects of MLC901 on tau phosphorylation
18.	Sex and the treatment effect in the Chinese Medicine NeuroAiD Efficacy on Stroke recovery (CHIMES) trial
19.	MLC901 Favors Angiogenesis and Associated Recovery after Ischemic Stroke in Mice
20.	Efficacy of MLC601 on functional recovery after stroke: A systematic review and meta-analysis of randomized controlled trials
21.	Positive effects of the traditional Chinese medicine MLC901 in cognitive tasks
22.	CHInese medicine NeuroAiD efficacy on stroke recovery - Extension study (CHIMES-E): A multicenter study of long-term efficacy
23.	Therapeutic Efficacy of Neuro AiD™ (MLC 601), a Traditional Chinese Medicine, in Experimental Traumatic Brain Injury
24.	Baseline characteristics and treatment response of patients from the Philippines in the CHIMES study
25.	MLC901, a Traditional Chinese Medicine induces neuroprotective and neuroregenerative benefits after traumatic brain injury in rats
26.	CHIMES-I: Sub-group analyzes of the effects of NeuroAiD according to baseline brain imaging characteristics among patients randomized in the CHIMES study
27.	Drugs from natural substances: Why study them in cerebral infarction
28.	NeuroAiD (MLC601) and amyloid precursor protein processing
29.	Chinese medicine NeuroAiD efficacy stroke recovery - Extension study (CHIMES-E study): An observational multicenter study to investigate the longer-term efficacy of NeuroAiD in stroke recovery
30.	NeuroAiD: Properties for neuroprotection and neurorepair
31.	Efficacy and safety of MLC601 (NeuroAiD®), a traditional chinese medicine, in poststroke recovery: A systematic review
32.	The NeuroAiD II (MLC901) in vascular cognitive impairment study (NEURITES) for the NEURITE investigators
33.	Cognitive enhancers (Nootropics). Part 3: Drugs interacting with targets other than receptors or enzymes. Disease-modifying drugs
34.	Burden of stroke in the Philippines
35.	Activation of ATP-sensitive potassium channels as an element of the neuroprotective effects of the Traditional Chinese Medicine MLC901 against oxygen glucose deprivation
36.	Alternative therapies for stroke treatment in Asia
37.	MLC901, a Traditional Chinese Medicine protects the brain against global ischemia
38.	NeuroAid (MLC601) versus piracetam in the recovery of post-infarct homonymous hemianopsia
39.	Safety profile of MLC601 (Neuroaid®) in acute ischemic stroke patients: A Singaporean substudy of the chinese medicine neuroaid efficacy on stroke recovery study
40.	Neuroprotective and neuroproliferative activities of NeuroAid (MLC601, MLC901), a Chinese medicine, in vitro and in vivo
41.	A double-blind, placebo-controlled, randomized phase II pilot study to investigate the potential efficacy of the traditional Chinese medicine neuroaid (MLC 601) in enhancing recovery after stroke (TIERS)
42.	A double-blind, placebo-controlled, randomized, multicenter study to investigate CHInese Medicine Neuroaid Efficacy on Stroke recovery (CHIMES Study)
43.	Neuroaid in stroke recovery
44.	Danqi Piantan Jiaonang does not modify hemostasis, hematology, and biochemistry in normal subjects and stroke patients
**Field of Biochemistry, Genetics and Molecular Biology**	
1.	A Long-term Study of NeuroAid (MLC601, MLC901) in Patients with Alzheimer's Disease; An Extension 8- year Follow-up Study
2.	Antiepileptic Effect of Neuroaid on Strychnine-Induced Convulsions in Mice
3.	Clathrin-nanoparticles deliver BDNF to hippocampus and enhance neurogenesis, synaptogenesis and cognition in HIV/neuroAIDS mouse model
4.	The Effect of NeuroAid (MLC901) on Cholestasis-Induced Spatial Memory Impairment with Respect to the Expression of BAX, BCL-2, BAD, PGC-1α and TFAM Genes in the Hippocampus of Male Wistar Rats
5.	Effects of morphine and NeuroAid on the expression levels of GluN2A and GluN3A in the hippocampus and striatum of rats
6.	The neuroprotective effect of NeuroAid on morphine-induced amnesia with respect to the expression of TFAM, PGC-1α, ΔfosB and CART genes in the hippocampus of male Wistar rats
7.	Effect of cholestasis and NeuroAid treatment on the expression of Bax, Bcl-2, Pgc-1α and Tfam genes involved in apoptosis and mitochondrial biogenesis in the striatum of male rats
8.	Intracyctic papillary carcinoma of the breast: Report of a rare case and literature review
9.	A positive correlation exists between neurotrauma and TGF-β1-containing microglia in rats
10.	A pharmacogenomic profile of human neural progenitors undergoing differentiation in the presence of the traditional Chinese medicine NeuroAiD

*Documents by affiliation*

**Figure 8.**
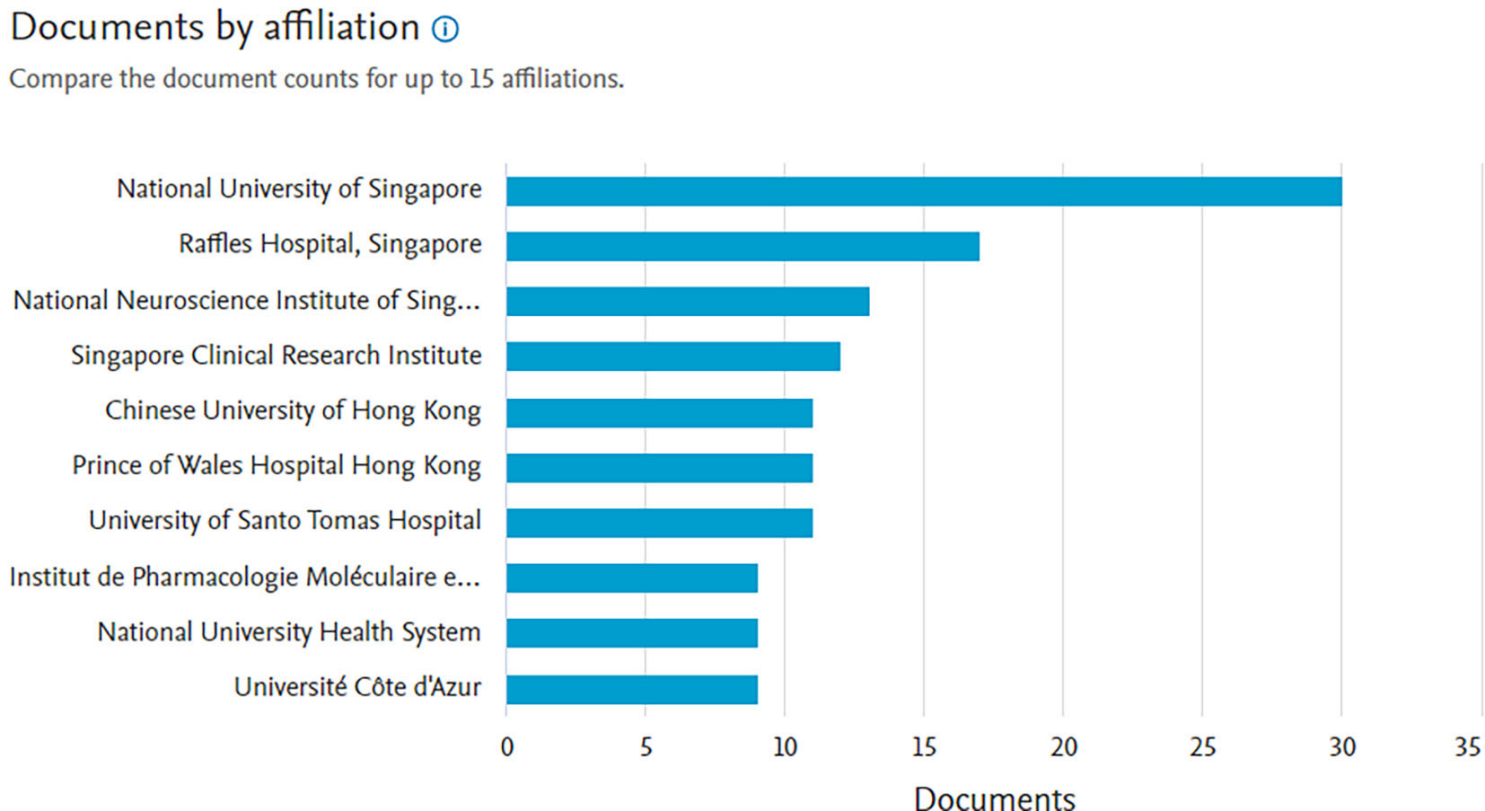
Documents by affiliation.

Based on Figure 8, in first place, the producer of the most documents is affiliated with the National University of Singapore with 30 documents, next in second place is affiliated with Raffkes Hospital, Singapore with 17 documents and next is the National Neuroscience Institute of Singapore with 13 documents.

*Network visualization*

**Figure 9.**
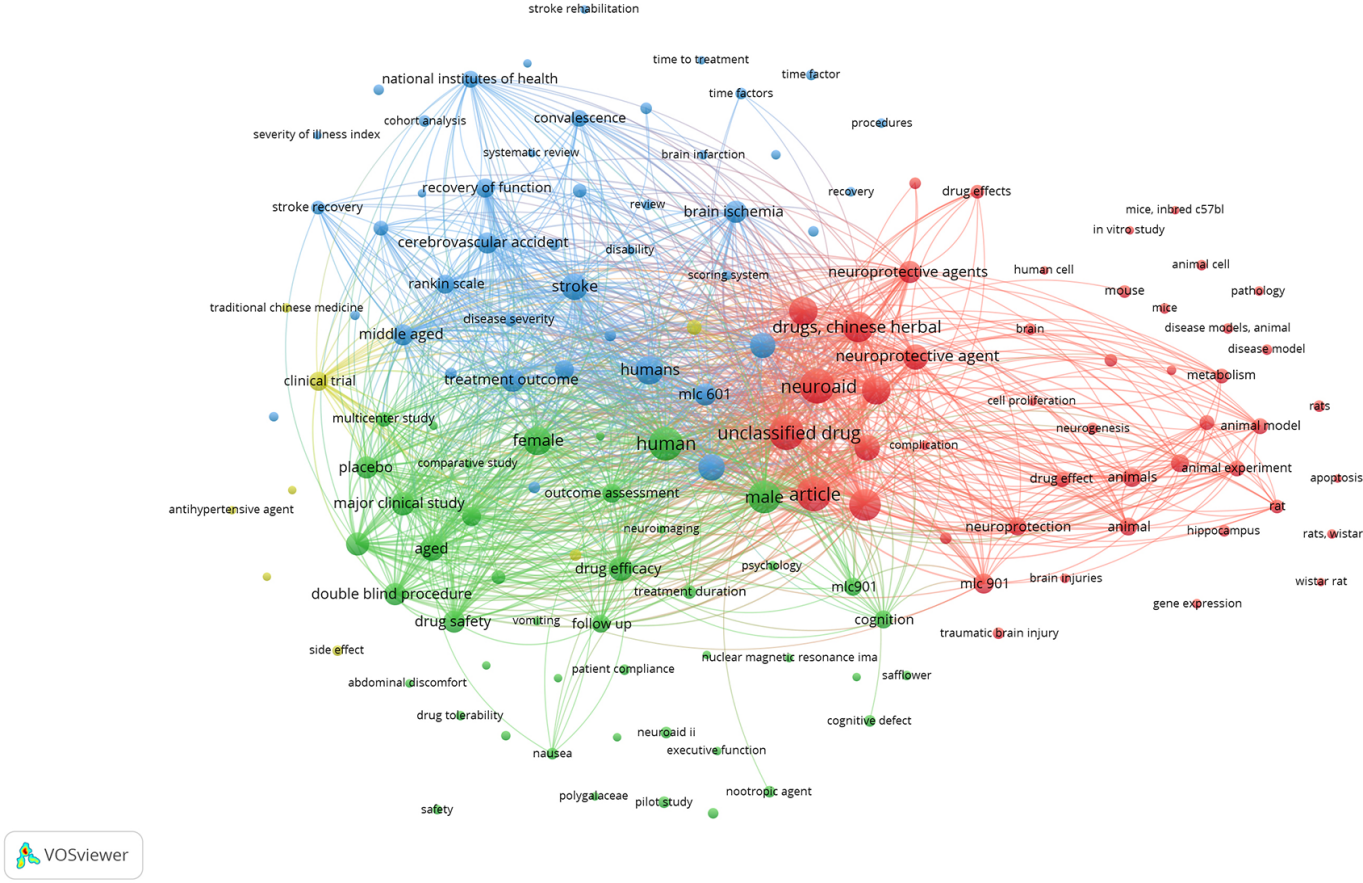
Network visualization.

Based on Figure 9, it can be seen that the areas studied are still not related to other areas that are divided into edges. That field is: stroke rehabilitation, time to treatment, time factor, procedures, severity of illness index, recovery, in vitro study, mice inbred c57bl, animal cell, mice, disease models animal, pathology, disease model, rats, apoptosis, rats wistar, wistar rat, gene expression, traumatic brain injury, safflower, cognitive defect, nuclear magnecti resonance imaging, neuroaid ii, executive function, nootropic agent, polygalacea, pilot study, drug tolerability, abdominal discomfort, safety, side effect, antihypertensive agent, traditional chinese medicine, brain injuries, hippocampus, cohort analysis, systematic review, brain infarction, brain, and human cell.

*Overlay visualization of scopus, database using vosviewer*

**Figure 10.**
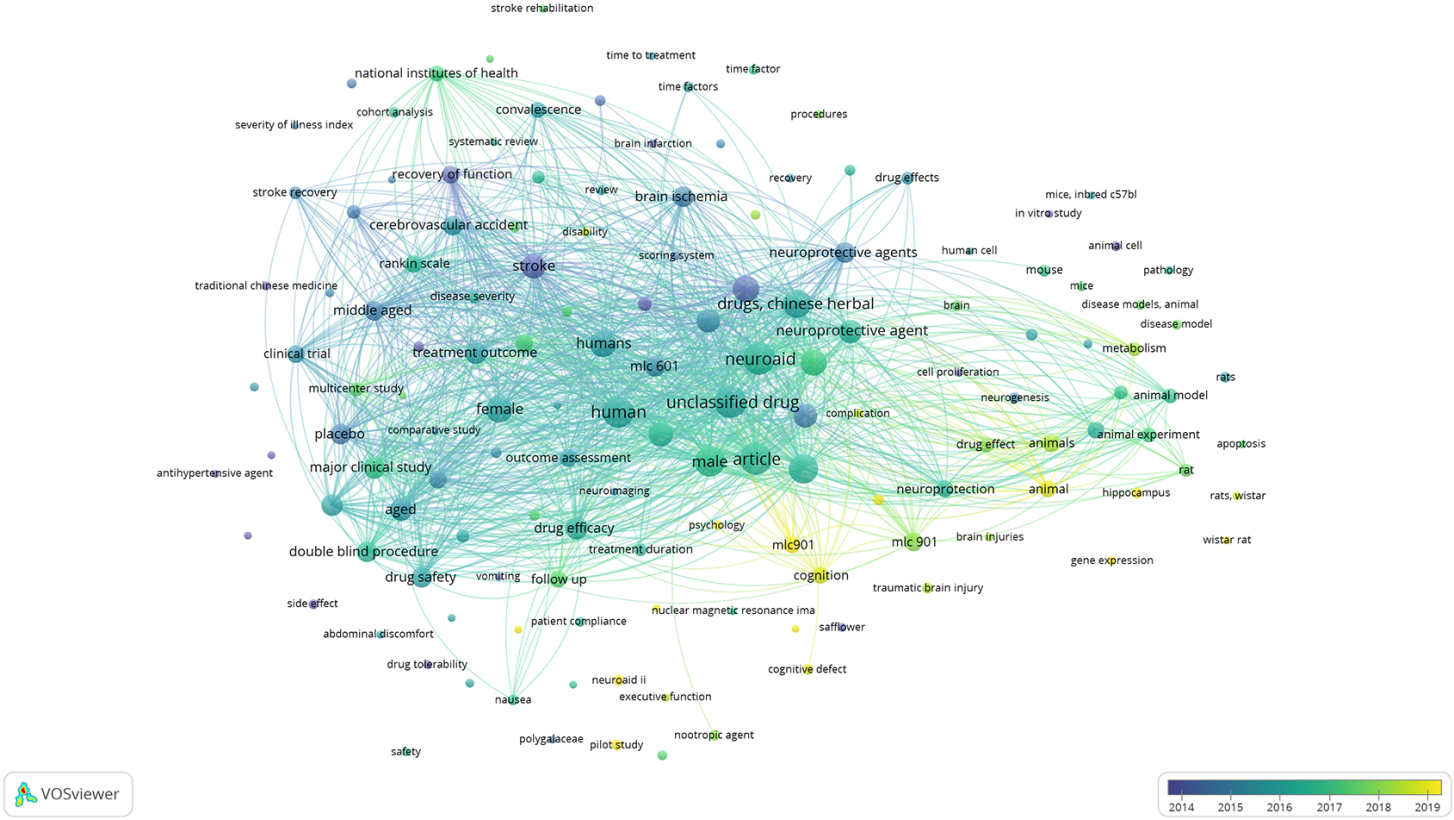
Overlay visualization of scopus, database using Vosviewer.

Based on Figure 10, in the overlay visualization, it appears that the keywords that are being researched a lot approaching 2019 are the parts colored yellow, namely: mic901, animal, pscychology, hippocampus, pilot study, cognition, wistar rat, disability, and neuroaid ii.

*Density visualization*

**Figure 11.**
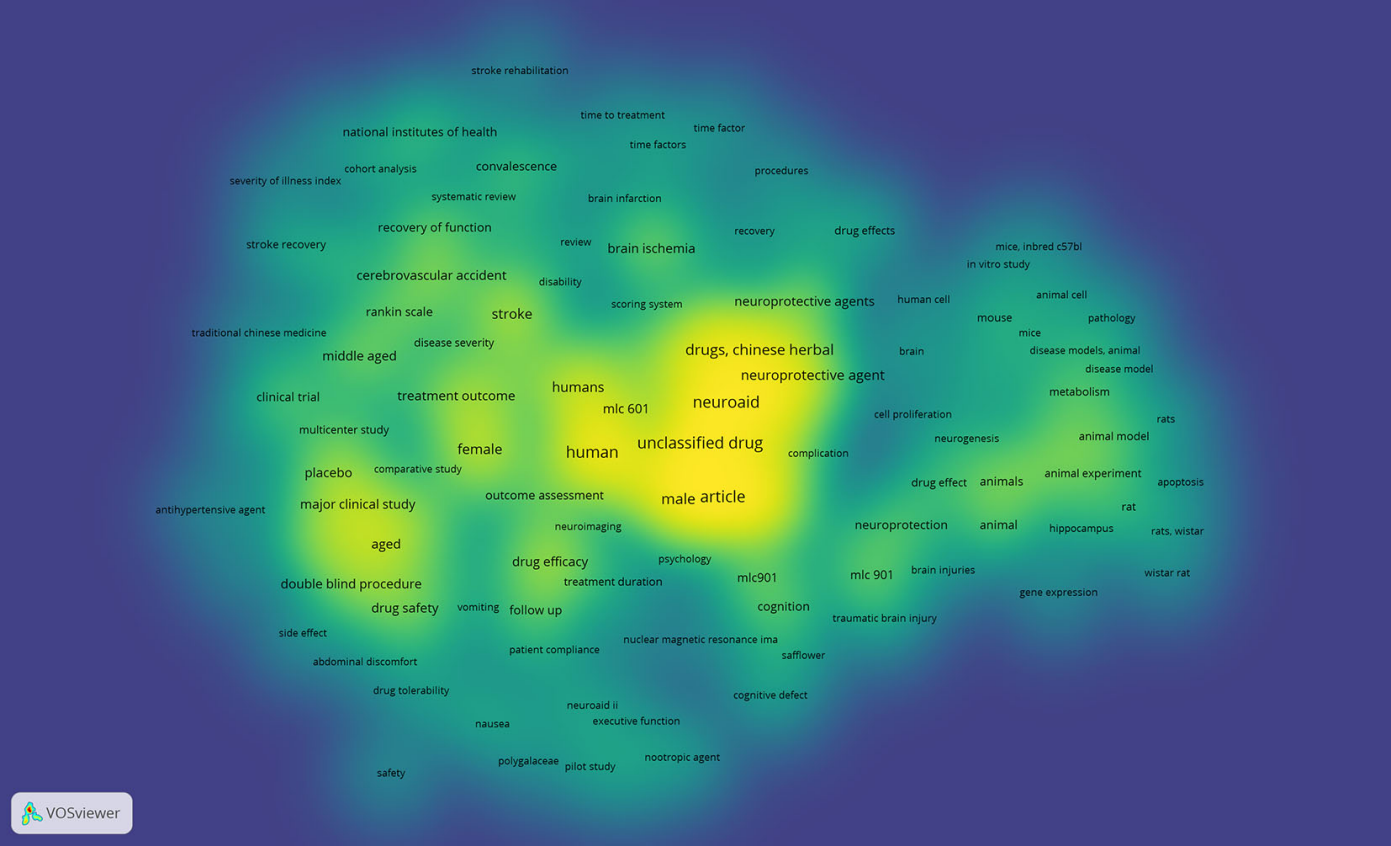
Density visualization.

Based on Figure 11, in the visual circulation density, it appears that the part that is already saturated with research is yellow, while the part that is not yet saturated is slightly yellow and dominantly green, namely keywords: stroke rehabilitation, time to treatment, time factor, time factors, procedures, recovery, drug effects, mice inbredc57bl, in vitro study, animal cell, mouse, mice, pathology, cell proliferation, rats, apoptosis, rat, rats wistar, hippocampus, wistar rat, gene expression, neurogenesis, safflower, cognitive defect, nuclear magnetic resonance ima, patient compliance, neuroaid ii, executive function, nootropic agent, pilot study, polygalaceae, naursea, safety, drug tolerability, abdominal discomfort, vomiting, side effect, antihypertensive agent, traditional chinese medicine, stroke recovery, severity of illness index, and cohort analysis.

*Thematic map*

**Figure 12.**
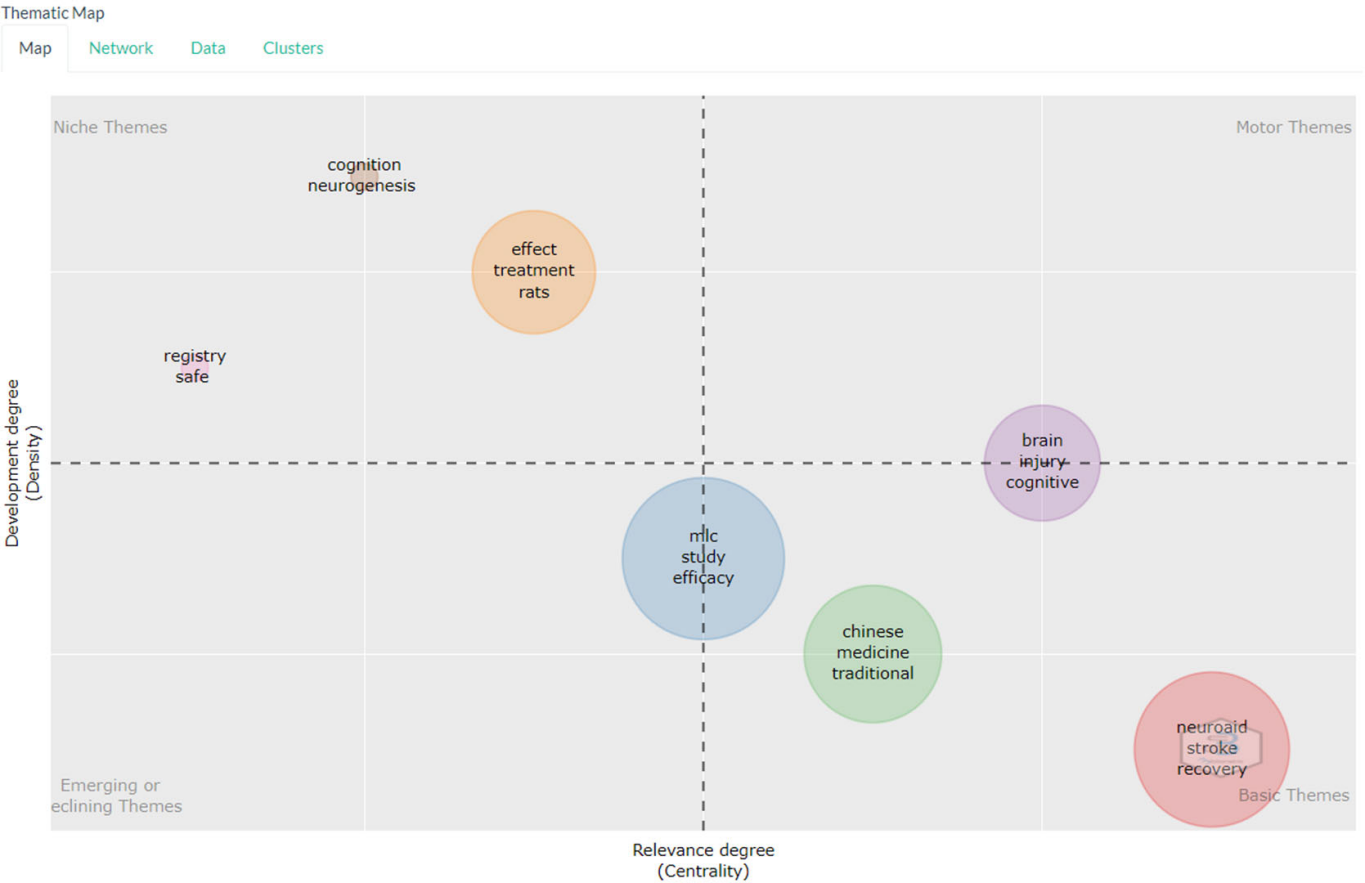
Thematic map.

Based on Figure 12, in the niche section there are the keywords cognition, neurogenesis then the keywords effect, treatment, rats then the keywords registry, safe.

*Thematic evolution*

**Figure 13.**
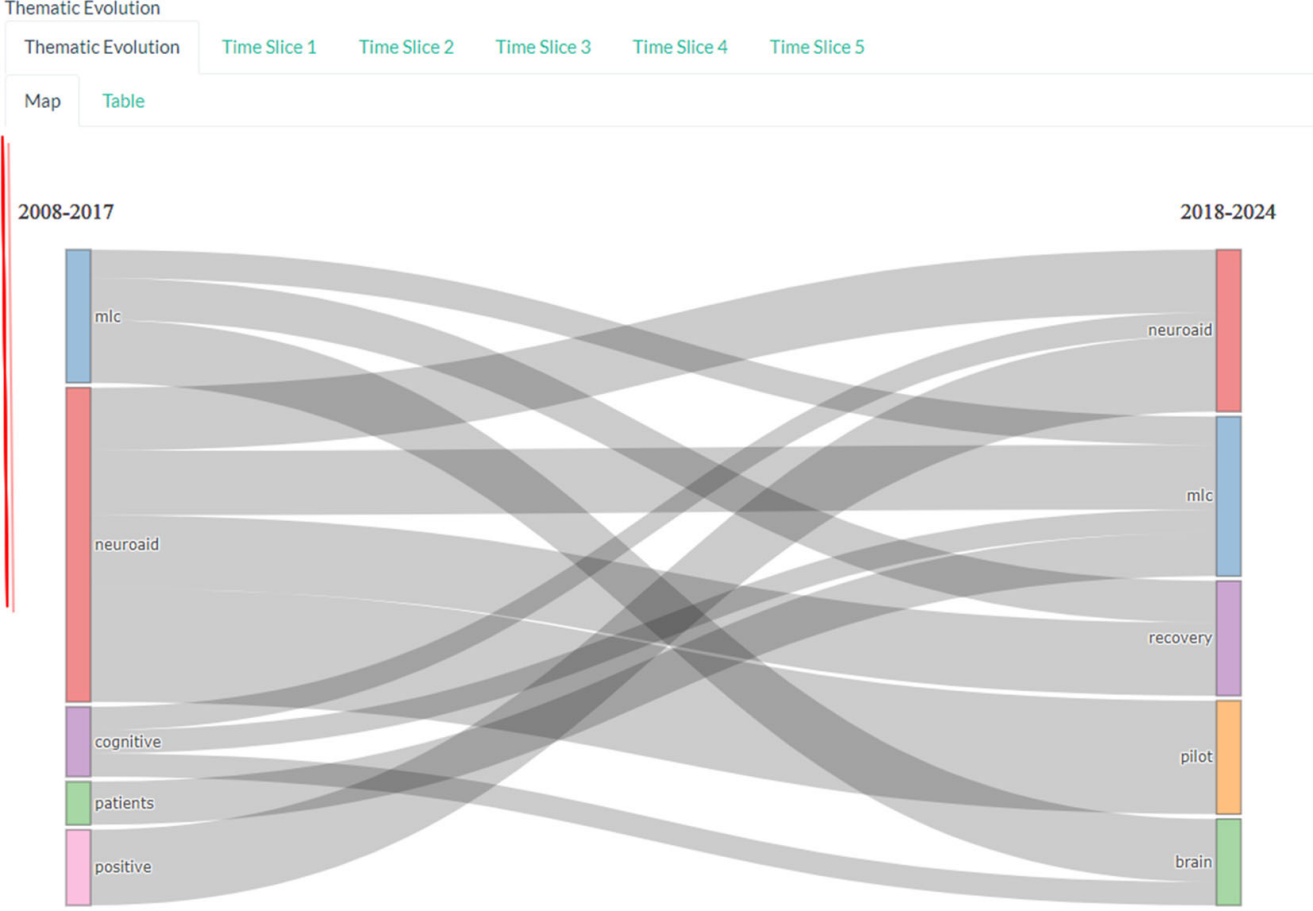
Thematic evolution.

Based on Figure 13, there was an evolution of changes in themes in research in 2008-2017 with the keywords mlc, neuroaid, cognitive, patients, and positive. The theme then changed in 2018-2024 to neuroaid, mlc, recovery, pilot and brain.

*Topic dendogram*

**Figure 14.**
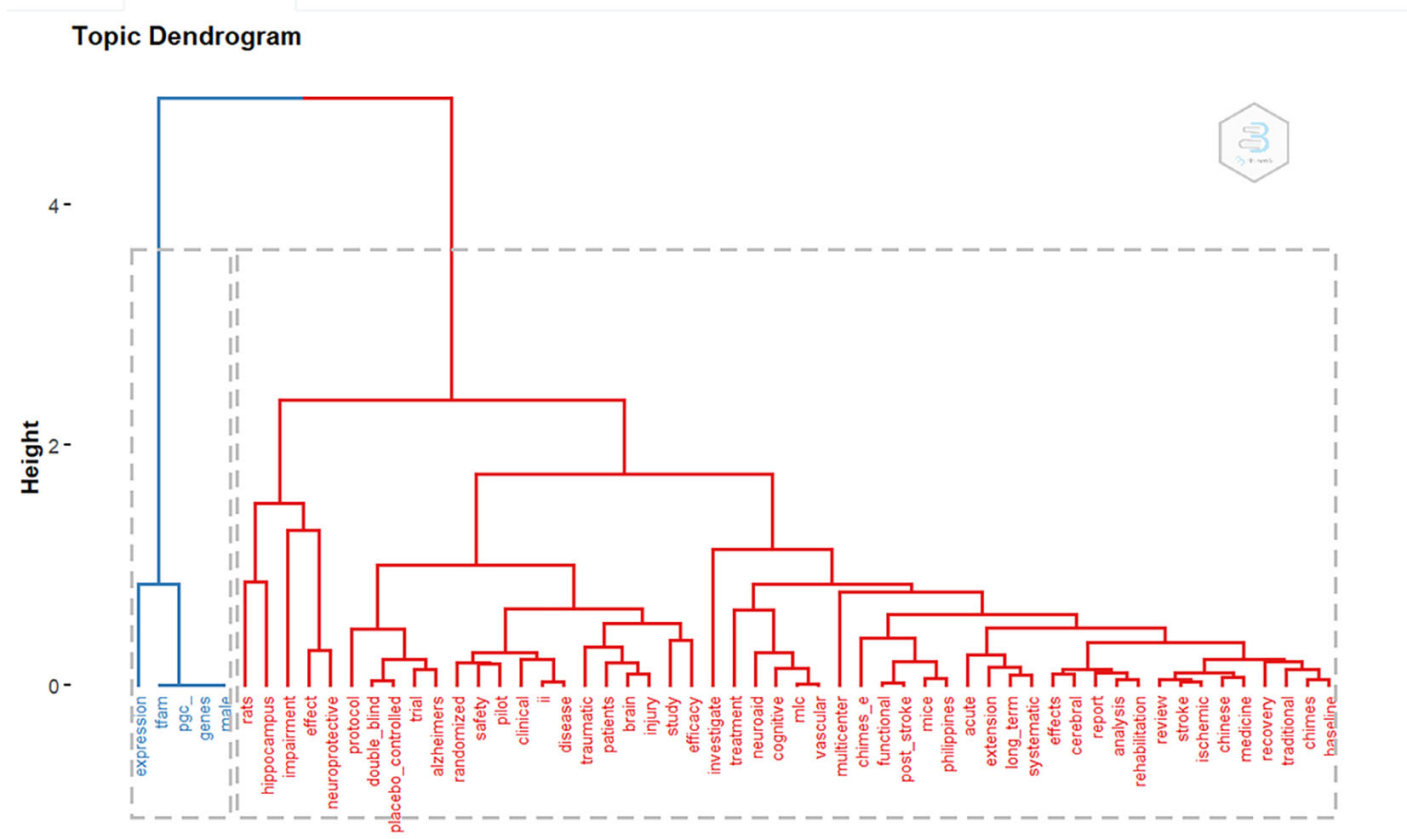
Dendogram.

Based on Figure 14, there are 2 large clusters based on keywords. There are 2 clusters of blue and red.

**Table 2.** Various research documents on the use of neuroaid.

Title	Document	Reference No.
Hemorrhagic stroke	2	^1^^,^^4^
Expression of bax Bcl-2 pgc-1alpha Tfam gene	3	^12^^,^^19^^,^^20^
Spinal cord injury	2	^9^^,^^21^
Vascular dementia	2	^10^^,^^22^
Parkinson	1	^23^
Traumatic brain injury	8	^3^^,^^24^^–^^30^
Cognitive impairment	5	^31^^–^^35^
Hypoxic ischemic brain injury	1	^36^
Nootropics	1	^37^
Epilepsy	1	^38^
Expression GluN2A	1	^39^
Cardioprotective	1	^40^
Alzheimer's disease	1	^41^
Tau phosphorilation	1	^42^
Amyloid precursor protein processing	2	^43^^,^^44^
Ischemic stroke	12	^2^^,^^45^^–^^55^
Neuroprotective	19	^56^^–^^74^
Review article	16	^5^^,^^7^^,^^8^^,^^75^^–^^87^

### Qualitative analysis

*Word cloud*

**Figure 15.**
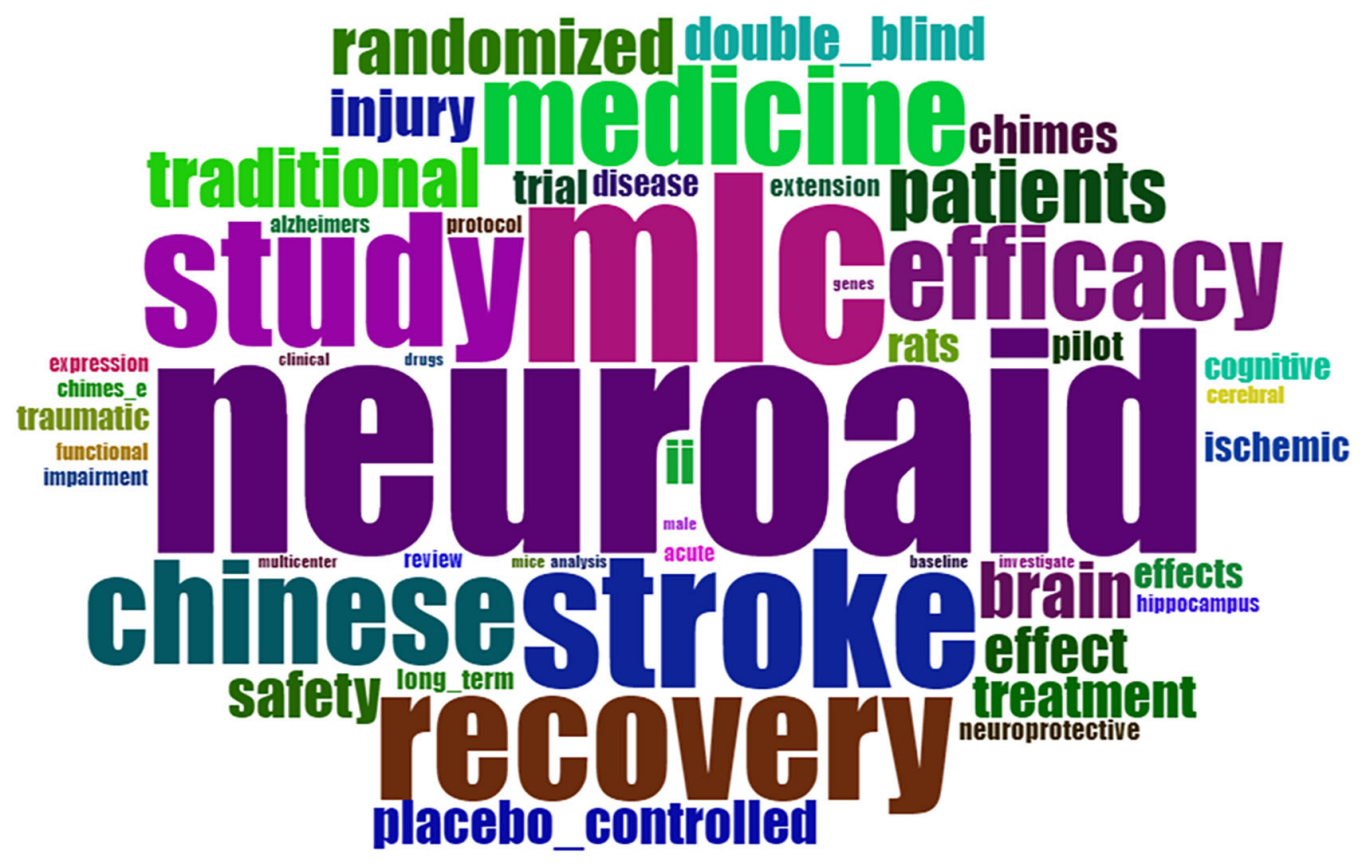
World cloud.

Based on Figure 15, word cloud shows that the dominance in the document is neuroaid, mlc, stroke, recovery, and study.

## Discussion

The niche theme in this study represents a more specific and perhaps less explored area of research in the current literature. For example, keywords such as “cognition” and “neurogenesis” may indicate that there is ongoing research into how neuroaid may affect cognitive function and the growth of new nerve cells. Meanwhile, keywords like “treatment,” “rats,” and “registry safe” may indicate that there is ongoing research into Neuroaid’s use in treatment, testing in mice, and its safety.

The motor theme includes keywords such as “brain,” “injury,” and “cognitive,” which may indicate that there is a lot of research that has been done on how Neuroaid can aid in recovery from brain injury and improvement of cognitive function. Other keywords such as “MLC,” “study,” “efficacy,” “Chinese,” “medicine,” “traditional,” “neuroaid,” “stroke,” and “recovery” indicate that there is extensive research on the efficacy of Neuroaid as a Traditional Chinese medicine in stroke recovery.

Themes that emerged or decreased were “MLC,” “study,” and “efficacy.” This suggests that research focus may be shifting in this direction or may indicate that research in this area may be declining. For example, if there is a decline in research on the efficacy of Neuroaid, this may indicate that researchers may be switching to another drug or therapy.

However, it is important to note that the results from this study may change if different keywords are used. This means that the interpretation of this study should be done with caution, considering that the results may be different if different keywords are used. For example, if the keyword “rehabilitation” is used instead of “recovery,” the results may be very different.

Additionally, this bibliometric study is new and uses document data from www.scopus.com. Therefore, the results may not reflect the entire literature available outside of this data source. For example, if there is research published in a journal that is not indexed in Scopus, it will not be included in this study. Nonetheless, this study provides valuable insight into current research trends and potential future research directions in the field of Neuroaid.

Neuroids, which were originally used to treat ischemic stroke, have now been used to treat hemorrhagic stroke, intelligent spinal injury, vascular dementia, Parkinson’s, traumatic brain injury, cognitive impairment, hypoxic ischemic brain injury, epilepsy, cardioprotective, and Alzheimer’s. With this bibliometric study, it is hoped that it can increase clinical insight so that neuroids can be included in recommendations and various expert consensuses in fields other than stroke.

## Conclusion

The study focuses on specific research areas and may have limited research on the topic. Key terms include “cognition” and “neurogenesis,” which indicate that neuroaid can influence cognitive function and new behaviors. Key terms include “treatment,” “rats,” and “registry safe,” which indicate that Neuroaid can help in stroke prevention and cognitive function enhancement. Key terms include “MLC,” “study,” and “efficacy,” which indicate that the focus of the study may be limited or irrelevant. However, the study’s findings may differ if different research bodies are used. Interpretation should be done with caution, considering the differences between the two groups. The study also uses bibliometric data from Scopus.com, which may not cover all available literature. Despite this, the study provides valuable insights into the research direction and potential areas of research in neuroscience. Neuroids are used for stroke prevention, hemorrhagic stroke, spinal cord injuries, vascular dementia, Parkinson’s, traumativ brain injury, cognitive impairment, hypoxic ischemic brain injury, epilepsi, cardioprotective, and Alzheimer’s. The bibliometric study can help create a consensus on neuroids for stroke treatment.

### Author contribution

AYS conducts research, gathers data, performs statistical analysis, and produces discussions and conclusions, RV, TDS and DAYS editing.

## Data Availability

Figshare: Beyond stroke therapy, neuroaid (a chinese herbal) has an effect on cognition and neurogenesis, a bibliometric study, https://doi.org/10.6084/m9.figshare.25943860.v1. ^88^ Figshare: PRISMA-ScR checklist: Beyond stroke therapy, neuroaid (a chinese herbal) has an effect on cognition and neurogenesis, a bibliometric study: https://doi.org/10.6084/m9.figshare.25943869.v1. ^89^ Data are available under the terms of the Creative Commons Attribution 4.0 International license (CC-BY 4.0).

## Software availability

VOSviewer software is an open-access tool that can be used as a cost-effective method for any scientometric analysis. ^90^
