# Supplementary material for: Beyond stroke therapy, neuroaid (a chinese herbal) has an effect on cognition and neurogenesis, a bibliometric study
Source: F1000Res. 2025 Sep 18;13:799. Originally published 2024 Jul 16. [Version 2] doi: 10.12688/f1000research.152581.2 (PMC12476539; doi:10.12688/f1000research.152581.2)
Supplement: Peer review history

# Reviewer report: Reviewer response for version 2

**DOI:** 10.5256/f1000research.188303.r415318

**Published:** 2025-09-27

**Authors:** Christopher Li Hsian Chen

**Affiliations:** 1 National University of Singapore, Singapore, Singapore

I have reviewed the article and have no additional comments. I approve it.

Are the rationale for, and objectives of, the Systematic Review clearly stated?

Yes

Is the statistical analysis and its interpretation appropriate?

Partly

If this is a Living Systematic Review, is the ‘living’ method appropriate and is the search schedule clearly defined and justified? (‘Living Systematic Review’ or a variation of this term should be included in the title.)

Not applicable

Are sufficient details of the methods and analysis provided to allow replication by others?

No

Are the conclusions drawn adequately supported by the results presented in the review?

Partly

Reviewer Expertise:

Translational medicine, Memory and Aging, Stroke, Clinical Trials, Neuroscience

I confirm that I have read this submission and believe that I have an appropriate level of expertise to confirm that it is of an acceptable scientific standard.

# Reviewer report: Reviewer response for version 1

**DOI:** 10.5256/f1000research.167360.r402766

**Published:** 2025-09-16

**Authors:** Christopher Li Hsian Chen

**Affiliations:** 1 National University of Singapore, Singapore, Singapore

The article “Beyond stroke therapy, NeuroAiD (a Chinese herbal) has an effect on cognition and neurogenesis: a bibliometric study” presents the first bibliometric analysis of the research landscape on NeuroAiD (MLC601/MLC901), a traditional Chinese herbal formulation originally developed for stroke recovery. NeuroAiD has been the subject of increasing investigation in preclinical and clinical studies, not only in post-stroke rehabilitation but also in relation to cognition, neurogenesis, and potential applications in conditions such as Alzheimer’s disease, traumatic brain injury, and other neurodegenerative disorders.

Key Findings and Conclusions

 The results confirm a steady growth of NeuroAiD-related publications, with Singapore and associated research groups being especially prominent contributors. Stroke therapy remains the dominant research theme, but clusters around “cognition” and “neurogenesis” have emerged, reflecting diversification of research interests. The most cited works include clinical trials (such as CHIMES) and systematic reviews, while preclinical studies also contribute to thematic diversity. The authors conclude that   research is expanding beyond its initial focus on stroke recovery to broader neurological and cognitive contexts. They further suggest that bibliometric findings may support the development of consensus recommendations and highlight promising directions for future clinical investigation.

Novelty and Interest

 The article is novel in being the first bibliometric synthesis of NeuroAiD research, filling an identifiable gap in the literature. By systematically mapping research output, it provides useful insights for clinicians, researchers, and policy-makers interested in complementary and integrative approaches to neurorehabilitation. The visualizations help reveal collaboration networks and research trajectories, which may guide future scientific planning.

Limitations as a Scientific Publication

 Despite its originality, the study has some methodological limitations.

 First, the reliance on a single database (Scopus) and a single keyword (“NeuroAiD”) risks missing relevant publications indexed under synonyms (e.g., MLC601, MLC901, Danqi Piantan Jiaonang). 

 Second, reproducibility is limited by the lack of detail on search strategy parameters, inclusion/exclusion criteria, and bibliometric software settings.

 Third, while the figures are generally clear, their interpretation occasionally overreaches by inferring therapeutic implications (e.g., stroke prevention, cognitive enhancement) from keyword clustering, which bibliometric mapping alone cannot establish. Finally, the conclusions would benefit from more cautious framing, emphasizing research trends rather than clinical efficacy.

Overall Assessment

 The study represents a timely and original contribution, offering a structured overview of how NeuroAiD research has developed and where it may be heading. Its main strength lies in novelty and descriptive clarity, but its scientific impact would be stronger if methodological transparency were enhanced and interpretive claims were more carefully aligned with the descriptive nature of bibliometric evidence.

**1. Are the rationale for, and objectives of, the Systematic Review clearly stated?**

 NeuroAiD is introduced as a traditional herbal formulation with applications in stroke recovery, cognition, and neurogenesis. The rationale is clear: while many clinical and preclinical studies exist, no bibliometric mapping has been performed.

Strengths:

 - The gap is well identified: absence of bibliometric studies despite a large literature base.

 - Objectives to analyze research trends, contributors, and thematic evolution are explicit.

Limitations:

 - The introduction mixes pharmacological background with bibliometric rationale, which dilutes focus.

 - Objectives could be sharper, ideally set out as 1–2 bullet points rather than embedded in long paragraphs.

 - Stronger linkage to how bibliometric insights can inform pharmacology and clinical research strategies or policy directions would improve the rationale.

**2. Are sufficient details of the methods and analysis provided to allow replication by others?**

 The general workflow is presented (Scopus search → CSV/RIS export → analysis with Biblioshiny and VOSviewer), but key details are insufficient for full replication.

Strengths:

 - Database (Scopus), search field (TITLE-ABS-KEY), and timeframe (May 2024) are reported.

 - Analytical tools are clearly identified and commonly used in bibliometric research.

Limitations:

 - Only one keyword ('NeuroAiD') was used, excluding synonyms such as 'MLC601,' 'MLC901,' or 'Danqi Piantan Jiaonang.' On the other hand, a few publications listed in the References seem to be irrelevant to NeuroAiD (see for example Ref. 23, 31, 81, 82, 85)

 - No inclusion/exclusion criteria or language filters were reported.

 - Data were extracted by one reviewer, with no mention of error-checking or inter-rater reliability.

 - No reporting of threshold parameters in VOSviewer/Biblioshiny, making replication of maps impossible.

 - The dataset (92 documents) is not openly shared, which limits validation.

**3. Is the statistical analysis and its interpretation appropriate?**

 The analysis is descriptive (publication counts, keyword clustering, co-authorship networks) and appropriate for bibliometrics. However, interpretation extends beyond what the methods can support. Figures and visual displays are integral to the study, and their clarity and interpretation also require assessment.

Strengths:

 - Appropriate use of standard bibliometric techniques and visualization tools such as co-authorship networks, thematic mapping, and keyword clustering.

 - Figures (documents per year, factorial maps, thematic maps, word clouds) illustrate results effectively and make complex patterns more accessible.

 - Most figures are clearly titled and correctly captioned, adding descriptive value.

 - Identification of most cited papers, productive countries, and thematic clusters is valid and well aligned with bibliometric outputs.

Limitations:

 - Lack of detail on statistical thresholds (e.g., co-occurrence strength, minimum citation counts) reduces reproducibility.

 - Some figures (network visualizations, density maps) are visually complex and not fully self-explanatory for readers unfamiliar with bibliometric software.

 - Legends sometimes assume prior knowledge of visualization conventions and could be made clearer.

**4. Are the conclusions drawn adequately supported by the results presented in the review?**

 The conclusions summarize observed research trends but sometimes make claims that exceed the descriptive scope of bibliometrics.

Strengths:

 - Accurately reflect publication growth, geographic distribution, and thematic emphasis on cognition and neurogenesis.

 - Some limitations (Scopus-only data) are acknowledged.

Limitations:

 - Overgeneralization to multiple conditions (Parkinson’s, epilepsy) is not justified.

 - Discussion underemphasizes citation clustering and network bias.

 - A more cautious conclusion would frame the study as mapping research directions, not   only evaluating therapeutic effectiveness.

**5. If this is a Living Systematic Review, is the ‘living’ method appropriate and is the search schedule clearly defined and justified?**

 This article is not designed or presented as a Living Systematic Review (LSR). The methodology corresponds to a one-time bibliometric analysis conducted on Scopus data in May 2024. Since it is not framed as a Living Systematic Review, criteria regarding search update schedules, predefined intervals, and ongoing maintenance do not apply. To avoid confusion, the authors could clarify explicitly in the methods or title that the work is a cross-sectional bibliometric study, not a living review.

Are the rationale for, and objectives of, the Systematic Review clearly stated?

Yes

Is the statistical analysis and its interpretation appropriate?

Partly

If this is a Living Systematic Review, is the ‘living’ method appropriate and is the search schedule clearly defined and justified? (‘Living Systematic Review’ or a variation of this term should be included in the title.)

Not applicable

Are sufficient details of the methods and analysis provided to allow replication by others?

No

Are the conclusions drawn adequately supported by the results presented in the review?

Partly

Reviewer Expertise:

Translational medicine, Memory and Aging, Stroke, Clinical Trials, Neuroscience

I confirm that I have read this submission and believe that I have an appropriate level of expertise to confirm that it is of an acceptable scientific standard, however I have significant reservations, as outlined above.

# Response

**Published:** 2025-09-16

**Authors:** ARMAN YURISALDI SALEH

**Affiliations:** Neurology, Universitas Pembangunan Nasional Veteran Jakarta, Jakarta, Special Capital Region of Jakarta, Indonesia

Thank you for the detailed and constructive feedback during the peer review process. 

**1. Are the rationale for, and objectives of, the Systematic Review clearly stated?**

 NeuroAiD is introduced as a traditional herbal formulation with applications in stroke recovery, cognition, and neurogenesis. The rationale is clear: while many clinical and preclinical studies exist, no bibliometric mapping has been performed.

Strengths:

 - The gap is well identified: absence of bibliometric studies despite a large literature base.

 - Objectives to analyze research trends, contributors, and thematic evolution are explicit.

Limitations:

 - The introduction mixes pharmacological background with bibliometric rationale, which dilutes focus.

 - Objectives could be sharper, ideally set out as 1–2 bullet points rather than embedded in long paragraphs.

 - Stronger linkage to how bibliometric insights can inform pharmacology and clinical research strategies or policy directions would improve the rationale.

**Introduction**

 NeuroAiD (MLC601/MLC901) is a traditional Chinese medicine formulation that has gained global attention for its role in post-stroke recovery. MLC601 contains nine herbal and five animal-derived components, while MLC901 is a simplified, animal-free version with comparable neuroprotective properties. Both have demonstrated efficacy in enhancing neurological recovery, promoting neurogenesis, and supporting cognitive function in conditions such as Alzheimer’s disease and traumatic brain injury.

 Despite the growing body of clinical and preclinical research, no bibliometric study has systematically mapped the scientific landscape surrounding NeuroAiD. Bibliometric analysis offers a powerful tool to:

- Identify research trends, thematic evolution, and collaboration networks.
- Inform future pharmacological research, clinical strategies, and policy development through evidence-based insights.

**Objectives**

 This study aims to:

1. Analyze publication trends, geographic distribution, and institutional contributions in NeuroAiD research from 2008 to 2024.
2. Identify the most prolific authors, journals, and subject areas associated with NeuroAiD.
3. Map thematic evolution and keyword clusters using bibliometric tools (Biblioshiny and VOSviewer).

**2. Are sufficient details of the methods and analysis provided to allow replication by others?**

 The general workflow is presented (Scopus search → CSV/RIS export → analysis with Biblioshiny and VOSviewer), but key details are insufficient for full replication.

Strengths:

 - Database (Scopus), search field (TITLE-ABS-KEY), and timeframe (May 2024) are reported.

 - Analytical tools are clearly identified and commonly used in bibliometric research.

Limitations:

 - Only one keyword ('NeuroAiD') was used, excluding synonyms such as 'MLC601,' 'MLC901,' or 'Danqi Piantan Jiaonang.' On the other hand, a few publications listed in the References seem to be irrelevant to NeuroAiD (see for example Ref. 23, 31, 81, 82, 85)

 - No inclusion/exclusion criteria or language filters were reported.

 - Data were extracted by one reviewer, with no mention of error-checking or inter-rater reliability.

 - No reporting of threshold parameters in VOSviewer/Biblioshiny, making replication of maps impossible.

 - The dataset (92 documents) is not openly shared, which limits validation.

 Thank you for the constructive feedback. We acknowledge that while the general workflow was outlined, several methodological details require clarification to ensure replicability.

- The search was conducted in Scopus using the TITLE-ABS-KEY field with the keyword “NeuroAiD” in May 2024. We recognize that excluding synonyms such as “MLC601,” “MLC901,” and “Danqi Piantan Jiaonang” may have limited the dataset and will address this in future updates.
- No language filters or inclusion/exclusion criteria were applied; this will be explicitly stated in the revised Methods section.
- Data extraction was performed by a single reviewer; we agree that inter-rater reliability and error-checking procedures should be described and will be incorporated.
- Threshold parameters used in VOSviewer and Biblioshiny (e.g., minimum co-occurrence, citation cut-offs) were not reported and will be added to support reproducibility.
- The dataset of 92 documents has now been made openly available via Figshare [https://doi.org/10.6084/m9.figshare.25943860.v1] to facilitate validation and transparency.

**3. Is the statistical analysis and its interpretation appropriate?**

 The analysis is descriptive (publication counts, keyword clustering, co-authorship networks) and appropriate for bibliometrics. However, interpretation extends beyond what the methods can support. Figures and visual displays are integral to the study, and their clarity and interpretation also require assessment.

Strengths:

 - Appropriate use of standard bibliometric techniques and visualization tools such as co-authorship networks, thematic mapping, and keyword clustering.

 - Figures (documents per year, factorial maps, thematic maps, word clouds) illustrate results effectively and make complex patterns more accessible.

 - Most figures are clearly titled and correctly captioned, adding descriptive value.

 - Identification of most cited papers, productive countries, and thematic clusters is valid and well aligned with bibliometric outputs.

Limitations:

 - Lack of detail on statistical thresholds (e.g., co-occurrence strength, minimum citation counts) reduces reproducibility.

 - Some figures (network visualizations, density maps) are visually complex and not fully self-explanatory for readers unfamiliar with bibliometric software.

 - Legends sometimes assume prior knowledge of visualization conventions and could be made clearer.

 Thank you for your constructive feedback. We agree that the analysis used is descriptive and in line with the bibliometric approach. Visualizations such as thematic maps, word clouds, and collaboration networks have been used to clarify publication patterns and research themes. However, we acknowledge that the interpretation of some sections needs to be adjusted to ensure they do not exceed the capabilities of the methods used. In the future, we will add details regarding statistical thresholds (e.g., strength of co-occurrence and minimum citation count) and clarify the legends and explanations of the visualizations to make them more understandable for the general reader.

**4. Are the conclusions drawn adequately supported by the results presented in the review?**

 The conclusions summarize observed research trends but sometimes make claims that exceed the descriptive scope of bibliometrics.

Strengths:

 - Accurately reflect publication growth, geographic distribution, and thematic emphasis on cognition and neurogenesis.

 - Some limitations (Scopus-only data) are acknowledged.

Limitations:

 - Overgeneralization to multiple conditions (Parkinson’s, epilepsy) is not justified.

 - Discussion underemphasizes citation clustering and network bias.

 - A more cautious conclusion would frame the study as mapping research directions, not   only evaluating therapeutic effectiveness.

 Thank you for the thoughtful feedback. We acknowledge that our conclusions may have extended beyond the descriptive scope of bibliometric analysis. In response, we have revised the conclusion to emphasize that this study primarily maps research directions and thematic trends in NeuroAiD-related literature, rather than evaluating therapeutic efficacy. While we noted emerging interest in conditions such as Parkinson’s and epilepsy, we now clarify that these references reflect exploratory research trajectories rather than established clinical applications. We also recognize the limitations of Scopus-only data and have added a note on potential citation clustering and network bias. These adjustments aim to align our interpretations more closely with the methodological boundaries of bibliometric research.

**5. If this is a Living Systematic Review, is the ‘living’ method appropriate and is the search schedule clearly defined and justified?**

 This article is not designed or presented as a Living Systematic Review (LSR). The methodology corresponds to a one-time bibliometric analysis conducted on Scopus data in May 2024. Since it is not framed as a Living Systematic Review, criteria regarding search update schedules, predefined intervals, and ongoing maintenance do not apply. To avoid confusion, the authors could clarify explicitly in the methods or title that the work is a cross-sectional bibliometric study, not a living review.

 Thank you for the comment. We clarify that this study is designed as a systematic review with a bibliometric approach. Although it does not follow the “living” review format, it systematically identifies, analyzes, and synthesizes published literature on NeuroAiD using predefined inclusion criteria and a transparent search strategy. The data extraction and analysis were conducted in accordance with established bibliometric review standards. We have updated the Methods section to explicitly state that this is a systematic, cross-sectional bibliometric review rather than a Living Systematic Review.

 Best Regard

 Arman

# Reviewer report: Reviewer response for version 1

**DOI:** 10.5256/f1000research.167360.r305119

**Published:** 2024-08-02

**Authors:** Ping-Chung Leung

**Affiliations:** 1 The Chinese University of Hong Kong, Hong Kong, Hong Kong

Stroke is a common debilitating disease with yet no standard treatment to improve the physical outcomes and mental disturbances during the rehabilitation period.  Use of Herbal medicine is yet a controversial form of remedy not widely accepted.  The use of substances of animal origin is another worrying component.  Authors rightly completed a thorough search for the support of using Euro Aid.  Apparently, most of the references originate from the CHIMES study.  Authors could discuss, via the literature reviews, all the points raised above.

Are the rationale for, and objectives of, the Systematic Review clearly stated?

Yes

Is the statistical analysis and its interpretation appropriate?

Yes

If this is a Living Systematic Review, is the ‘living’ method appropriate and is the search schedule clearly defined and justified? (‘Living Systematic Review’ or a variation of this term should be included in the title.)

Yes

Are sufficient details of the methods and analysis provided to allow replication by others?

Yes

Are the conclusions drawn adequately supported by the results presented in the review?

Yes

Reviewer Expertise:

Research on traditional medicine

I confirm that I have read this submission and believe that I have an appropriate level of expertise to confirm that it is of an acceptable scientific standard.

# Response

**Published:** 2024-08-03

**Authors:** ARMAN YURISALDI SALEH

**Affiliations:** Neurology, Universitas Pembangunan Nasional Veteran Jakarta, Jakarta, Special Capital Region of Jakarta, Indonesia

Thank you to the reviewers for their kindness in providing feedback and suggestions.
